# Therobotat the sideof the nurse: present situation and prospects

**DOI:** 10.1186/1824-7288-41-S1-A8

**Published:** 2015-09-24

**Authors:** LauraDel Mastro, Laura Plevani, Giacomo Cavallaro, Fabio Mosca

**Affiliations:** 1 Neonatal Intensive Care Unit, Department of Clinical Sciences and Community Health, Fondazione IRCCS Cà Granda, Ospedale Maggiore Policlinico, University of Milano, Italy

## 

Patient safetyis a priority worldwide. According to the WHO medical errors involve 1 in 10 patients, providing damageor injury, including standing up to cause death [1]. Even the nurses can not refrain from protecting the patient as regards security, as explained in Art. 29 of the Code of ethics of nurses [2]. The complexity of the Neonatal Intensive Care Unit (NICU), the vulnerability of the neonatal population and the use of drugs “off-label” increase the risk of medical errors [3,4].

The literature reports that the risks of medical errors are related to the preparation of injectable drugs, quantified, insome cases, up to26.90% (?) of the doses somministrate [5].

A new robotic technology, called ivSTATION®(Health-Robotics) and specifically developed for the preparation of injectable ready to administration, was introduced in our NICU to simplify the therapeutic processin the preparation stage, to optimize resources and times and especially to enhance the security. ivSTATION® is able to reconstitute powdered drugs, to obtain dilutions specifications, to verify doses prepared before making them available and to provide them with a label containing all the information required. The therapy is prepared in an aseptic environment ISO 5equipped with HEPA filters 14. The unit is equipped with UV lamps that contribute to the microbiological control overnight. ivSTATION® provides total trace ability of information about each preparation produced, making it possible to extract all the time both data specific to a single preparation and general data to be used for statistical analysis. Once the drug is prepared, it appears on the medical record through a symbol, allowing the nurse to proceed with the implementation of the successive phases of the therapeutic process, in strict observance of safety rules. The integration of the robot in the preparation of the therapies, associated with the system of the therapeutic process with the computerized system “bar code”, allows a considerable decrease of the risk in both the preparation phase of identification of the patient, which are known to those where the errors are hardly detectable [6,7].

This technology is a useful tool for limiting the error and the niatrogenic clinical risk associated with medical therapy. The nurse, in charge of patients, has the opportunity, with the use of this technology, to spend more timenursing careproper.
